# Ergota as a Galactifuge

**Published:** 1876-04

**Authors:** C. Henri Leonard

**Affiliations:** Detroit, Mich.


### ERGOTA AS A GALACTIFUGE.

By C. HENRI LEONARD, M.D., Detroit, Mich.

Recently I had quite a novel experience in the use of ergota. The story, in short, is told in the caption of this article. Deeming, however, a little prolixity not unacceptable, I give you a somewhat brief *résumé* of the case.

Mrs. ——, aged 26, had a rather hurried, otherwise normal, confinement. On the third day the breasts were full; no fever or other abnormal symptom occurred. On the fourth and fifth days there was a free secretion of milk, so much so that the breasts were relieved by manipulation, the babe not being able to take it all. On the afternoon of the 5th day, my patient was doing so nicely that I gave her permission to sit up (bolstered) in bed a few moments. She exceeded my permission, and so held the babe, and "changed" it. She became quite tired, and before lying down a pretty sharp hæmorrhage (uterine) set in. I gave her twenty minims of the fluid extract of ergot, (Parke, Davis & Co.'s manufacture) combined with some simple adjuvants, twice during the evening, the hæmorrhage then ceasing. On the afternoon of the next day, after being guilty of the same imprudence as on the day before, the flowing again occurred, even more freely than before. I then placed her upon the same treatment, directing four doses daily, to be continued two or three days. She was thus getting about eighty minims of the ergot daily. On the second day of the taking of the drug the secretion of the milk was notably lessened. There was no fever, or other abnormal symptoms made manifest. On the third day the supply was so much lessened, that artificial feeding of the infant was necessary; both breasts being equally deficient in the amount of nutriment.

I was completely nonplussed at this, for my patient, otherwise, was doing as nicely as any woman could do; and in her former nursing period, the supply had, for a long time, exceeded the demand. However, I withdrew the ergot mixture; not because I *then* attributed the trouble to that, but more to satisfy her, as she attributed her trouble to the medicine. Upon a second thought I was inclined to side with the views of my patient, and believe the ergot to be at the bottom of the mischief. My reasoning was, that if it will stimulate the nonstriated fibres of the uterus, intestines, arteries and veins (M Vulpian) to contraction, thus diminishing the blood supply, why may it not, for the same reason, deprive the breasts of their usual amount of blood and thus lessen the throwing off of the milk cells, and diminish the amount of secretion? Further than this, the same action might be conceded as taking place in the walls of the secreting cellules and ducts of the mammæ, and so aiding in diminishing the milk supply—something of the same line of action that is manifest in checking certain watery diarrhœas, even though they (the intestinal discharges) may be more properly classed with transudation rather than secretion.

As a clinical proof of the truthfulness of my reasoning,

I would state that, in a few days, the milk was again secreted in its normal abundance, and the child (at this writing, about two months of age) receives all the nourishment it needs from its mother. There have been at no time any symptoms of fever, diarrhœa, pelvic tenderness, or anything of a suspicious character in the symptoms presented by the mother, save the one, hæmorrhage, which was readily stopped by the use of ergot.

Nothing in my library gave me any similar account of the action of this drug, neither could I learn, upon inquiry, that any of my friends had experienced the same results from its administration. Some days after this episode of mine, *The Richmond and Louisville Medical Journal* came to hand, and in an essay upon Ergot, by Dr. Hadra, of Texas, I find that he quotes Le Gendre as an authority for using the drug in galactorrhœa; but only refers to it incidentally. However, in the "Clinical and Pharmaceutical Department" of the same number of the above Journal I find the following *excerptum:* "During an epidemic of *secale cornutum*, in the district of Simbirski, Dr. J. Schtscherbinenkoff found that among the symptoms of ergot-poisoning was a diminution, or a complete arrest, of the secretion of milk in lactating women. The same result was found to occur in cows that had been fed on meal which contained ergot, or had been littered with carelessly threshed straw which still contained some affected ears. Dr. S. conceived the idea of employing it as a remedy in case of threatened abscess of the breast, and carried it out in many cases, with great advantage. Two multiparæ, who had suffered at each previous confinement from abscess of the breast, took some of the drug with the happiest result. He has also found the drug useful in cases of 'milk fever,' and also at the weaning of the child, whether at the normal or an earlier period."

This is the extent of the literature upon the subject, so far as I am acquainted with it. Certainly, if the action of the drug, as witnessed in my patient, is to be anything of a criterion of its reliability as a galactifuge, the customary belladonna, magnesiæ sulphas, and potassii iodidum treatment must be superseded in the treatment of many of the common ailments of our nursing women. Have any of the JOURNAL readers had any similar experience with the drug?

